# Does docosahexaenoic acid supplementation in term infants enhance neurocognitive functioning in infancy?

**DOI:** 10.3389/fnhum.2013.00774

**Published:** 2013-11-20

**Authors:** Alexandra E. Heaton, Suzanne J. Meldrum, Jonathan K. Foster, Susan L. Prescott, Karen Simmer

**Affiliations:** ^1^School of Paediatrics and Child Health, University of Western AustraliaPerth, WA, Australia; ^2^School of Psychology and Speech Pathology, Curtin Health Innovation Research Institute, Curtin UniversityPerth, WA, Australia; ^3^Neurosciences Unit, Western Australia Department of HealthPerth, WA, Australia; ^4^Telethon Institute for Child Health ResearchPerth, WA, Australia; ^5^Centre for Neonatal Research and Education, University of Western AustraliaPerth, WA, Australia

**Keywords:** neurocognitive, development, n-3 LC-PUFA, DHA, infant

## Abstract

The proposal that dietary docosahexaenoic acid (DHA) enhances neurocognitive functioning in term infants is controversial. Theoretical evidence, laboratory research and human epidemiological studies have convincingly demonstrated that DHA deficiency can negatively impact neurocognitive development. However, the results from randomized controlled trials (RCTs) of DHA supplementation in human term-born infants have been inconsistent. This article will (i) discuss the role of DHA in the human diet, (ii) explore the physiological mechanisms by which DHA plausibly influences neurocognitive capacity, and (iii) seek to characterize the optimal intake of DHA during infancy for neurocognitive functioning, based on existing research that has been undertaken in developed countries (specifically, within Australia). The major observational studies and RCTs that have examined dietary DHA in human infants and animals are presented, and we consider suggestions that DHA requirements vary across individuals according to genetic profile. It is important that the current evidence concerning DHA supplementation is carefully evaluated so that appropriate recommendations can be made and future directions of research can be strategically planned.

## LC-PUFA in the current human diet and the importance of DHA for early brain development

The omega-3 (*n*-3) long chain polyunsaturated fatty acid (LC-PUFA), docosahexaenoic acid (DHA; 22:6 *n*-3), is involved in several critical brain functions (Bradbury, 2011). DHA accretes within the brain during gestation and the first year of life during the “brain growth spurt” (Innis, 2008). During this time, the developing brain is sensitive to extreme variations in the supply of DHA (Karr et al., 2011). DHA accretion in the fetal brain can be influenced by maternal diet, DHA stores, placental transport, and genetic polymorphisms (McCann and Ames, 2010). Dietary DHA is obtained during the postnatal period via breast milk or infant formula. The concentration of DHA within breast milk can vary depending on maternal DHA stores and diet (Innis, 2008). The average DHA intake of Australian women during pregnancy and lactation is below global recommendations; this is likely to impact the provision of DHA to the offspring (Bourre, 2007; Meyer, 2011).

Formula fed infants in many countries, including Australia, may also be at risk of receiving insufficient dietary DHA, since the inclusion of pre-formed *n*-3 and *n*-6 LC-PUFAs (including DHA) in formula is not mandatory. It is argued that infants are capable of synthesizing *n*-3 and *n*-6 LC-PUFAs endogenously from their shorter chain precursors (Guesnet and Alessandri, 2011). However, there is considerable debate around human capacity and ability to synthesize DHA. Research has found single nucleotide polymorphisms (SNPs) in the fatty acid desaturase genes (FADS1 and FADS2) modulate individual capacity for LC-PUFA synthesis (Glaser et al., 2011). Subsequently, it is now recognized that dietary requirements for DHA and other LC-PUFAs may vary across the population and are somewhat dependent on individual genetic profile (Koletzko et al., 2008). The need for LC-PUFA supplementation in term infants therefore remains unknown. This article summarizes the major animal studies and clinical trials pertaining to fatty acid supplementation during infancy, and evaluates the current level of evidence for LC-PUFA supplementation in term infants.

**Figure 1:**
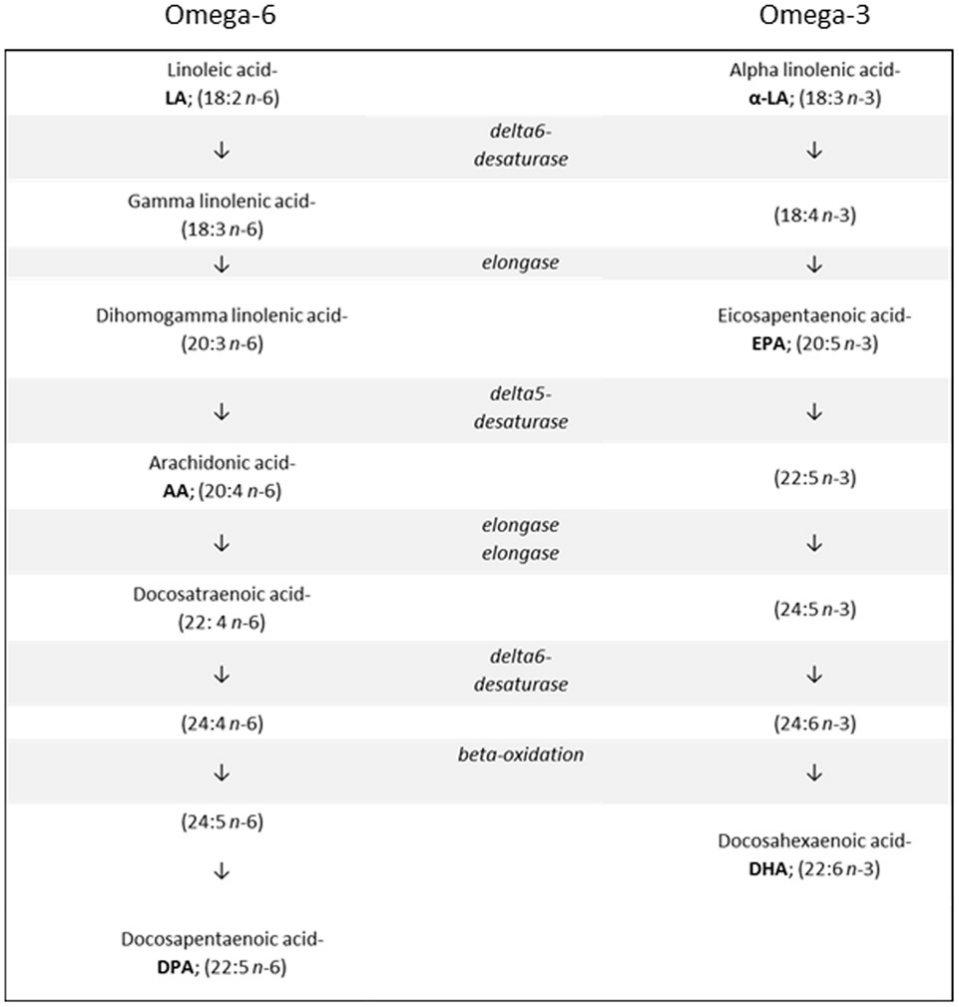
**The metabolic pathways involved in synthesizing n-6 and n-3 long chain polyunsaturated fatty acids from their respective shorter chain precursors**.

The *n*-3 and *n*-6 families are distinct groups of PUFAs important for human health, growth, and development. All PUFAs within the *n*-3 family are derived from alpha linolenic acid (α-LA; 18:3 *n*-3), while all *n*-6 PUFAs are derived from linoleic acid (LA; 18:2 *n*-6). (Figure 1) The (short chain) parent molecules of the *n*-3 and *n*-6 families, α–LA and LA, are described as “essential” fatty acids, since humans and other mammals cannot synthesize them endogenously (Kris-Etherton et al., 2000). The three most biologically active members of these families are the LC-PUFAs: DHA, eicosapentaenoic acid (EPA; 20:5 *n*-3) and arachidonic acid (AA; 20:4 *n*-6). These three fatty acids all carry out separate, complex functional roles in the body (McNamara and Carlson, 2006).

For Western nations, low dietary intake of EPA and DHA is a concern as these substances are not widely available in contemporary diets (Calder, 2012). There are few plant sources of DHA and EPA, and they are found almost exclusively in oily, cold water fish, fish oil supplements, breast milk, and supplemented infant formula. α–LA is available in oils such as canola and soybean, as well as in walnuts and flaxseed (Table 1). Converting α–LA into EPA and DHA is a variable and inefficient process; stable-isotope tracer studies have shown that dietary α-LA accounts for between 0.2 and 8% of EPA and <0.05 and 4% of DHA (Burdge and Calder, 2005; Plourde and Cunnane, 2007). Similarly, less than 0.1% of LA is converted into AA. Furthermore, conversion levels vary according to the intake of other fatty acids and the ratio of *n*-6: *n*-3 PUFAs consumed in the diet (Bokor et al., 2010). *n*-6 PUFA intakes in Western diets are typically high, supplying ~16 times more *n*-6 PUFA (including AA) than *n*-3 PUFA due to high intakes of beef, pork, poultry, wheat germ, and various cooking oils (Simopoulos, 2001b). In contrast, the diets of our paleolithic ancestors are thought to have contained roughly equal ratios of *n*-3: *n*-6 (Simopoulos, 2001a). Subsequently, there has been some debate as to whether modern diets contain sufficient quantities of *n*-3 fatty acids (in particular the *n*-3 LC-PUFAs, DHA and EPA) to support optimum health across the lifespan, especially during pregnancy and lactation (Eaton, 2000). It is possible that typical current levels of DHA intake are sufficient to achieve optimal neurocognitive functioning in childhood. This is the focus of the current review.

**Table 1:** **Common dietary sources of *n*-3 and *n*-6: short chain essential fatty acids (α-LA and LA) and long chain polyunsaturated fatty acids (DHA and AA) (Howe et al., 2006; Russo, 2009)**.

	**Omega-3 PUFA**		**Omega-6 PUFA**	
Dietary sources: EFA	α-LA	Vegetable oils: linseed and canola Flaxseed and walnut Fish: herring, salmon, and tuna Green leaves	LA	Vegetable oils: Corn, sunflower, and safflower Pork Walnut, peanut, and wheat Fish: herring, salmon, and tuna
Dietary sources: LC-PUFA	DHA & EPA	Fish: herring, salmon, trout, tuna, and fish oil supplements Breast milk	AA	Beef, pork, and poultry Whole-grain wheat

## DHA within the brain affects neuronal development

There have been several lines of evidence that suggest that LC-PUFAs (particularly DHA) are important for the function of the CNS at the cellular and neurobiological level. The concentration of DHA and other LC-PUFAs within the brain can alter the neuronal membrane fluidity and physical structure of neurons (Youdim et al., 2000). *n*-3 and *n*-6 LC-PUFAs are also involved in the production and activity of several neurotransmitters such as dopamine and serotonin (Zimmer et al., 2002; Aid et al., 2005; Chalon, 2006), affecting synaptic transmission and substrate binding to membrane receptors (Horrocks and Farooqui, 2004). Furthermore, DHA has been shown to affect neural functioning via its influence on gene expression in mammalian brain tissue (de Urquiza et al., 2000; Kitajka et al., 2004). The process of neurite outgrowth in hippocampal neurons is enhanced by DHA, which may in turn promote learning (Calderon and Kim, 2004) as the hippocampus is a critical brain region for memory formation (Rolls, 2008; Berger et al., 2012). DHA may also improve learning and memory through its role in the development of pre- and post-synaptic proteins which enable synaptic transmission and long-term potentiation (Cao et al., 2009). It is apparent that DHA plays numerous important biophysical roles in brain structure and function, and has the potential to influence neurocognitive development and subsequent performance (McNamara and Carlson, 2006; Parletta et al., 2013).

During the last trimester of gestation and for the first 18 months after birth, AA, and DHA are deposited within the cerebral cortex at a rapid rate (Martinez et al., 1974; Clandinin et al., 1980). As noted above, this stage of human development is known as the brain growth spurt (Martinez and Mougan, 1998), when neuronal development is particularly vulnerable to nutritional insufficiencies (Nyaradi et al., 2013). Further evidence from studies on young non-human primates has revealed that once brain DHA depletion has occurred it is physiologically difficult to reverse (Ikemoto et al., 2001).

## Dietary recommendations for DHA during pregnancy and the neonatal period

Considering the rapid accretion of DHA into the brain during the last trimester of pregnancy and into the first year of life, it is important to consider whether optimal DHA intake is occurring during this period. Numerous expert and government authorities worldwide agree that dietary DHA requirements are increased during pregnancy and lactation when a minimum of 200 mg of DHA per day is recommended (Van Elswyk and Kuratko, 2009). This dose can be achieved by eating 1–2 portions of fish per week or taking fish oil supplements (Koletzko et al., 2008). Recent figures indicate that 91% of Australian women are failing to meet DHA recommendations during pregnancy and lactation (Cosatto et al., 2010), with similar trends noted in many other Western countries (Meyer, 2011). In Australia, the median daily intake of DHA during pregnancy is ~96 mg per day, ranging from 8 to 632 mg per day across individuals (Cosatto et al., 2010). Since the dietary intake and maternal stores of DHA during pregnancy are known to be key determinants of infant blood DHA concentrations at birth (Bourre, 2007), low DHA consumption by women eating Western diets has prompted some concern for the neurological and neurocognitive development of their offspring (Rogers et al., 2013).

With respect to intake of DHA via breastfeeding, findings from the 2010 Australian National Infant Feeding Survey have revealed that while around 96% of Australian women initiate breastfeeding after birth, there is a rapid decline in breastfeeding rates during the weeks and months following birth (AIHW, 2011; Burns et al., 2012). Despite international recommendations for exclusive breastfeeding for the first 6 months postnatally (Kramer and Kakuma, 2001), in Australia an average of only 56% of women breastfeed for this recommended amount of time (AIHW, 2011).

Moreover, the concentration of DHA within women's breast milk appear to be decreasing over time (Makrides et al., 1995). A comprehensive analysis of human breast milk LC-PUFA compositions have revealed the current worldwide average DHA concentration is approximately 0.32% of total fatty acids (TFA) (Brenna et al., 2007). This corresponds to ~60 mg of DHA per day for the first 6 months postnatally, assuming an average breast milk intake of 750 mL per day (Cunnane et al., 2000). Analysis of Australian breast milk concentrations in 1981 and 1993 found that the amount of DHA decreased by 27% over this time period while concentrations of AA remained the same (Makrides et al., 1995). A similar decline in human milk DHA has been reported in a Canadian population (Innis, 2003). The decline in breast milk DHA may plausibly be explained by dietary shifts over time, the use of different FA analytical techniques (Makrides et al., 1995) or through differences in maternal FADS genotype across studies (Xie and Innis, 2008; Moltó-Puigmartí et al., 2010)—an issue that will be discussed further below.

Formula-fed infants may also be at risk of sub-optimal DHA supply during this period. DHA brain content has been analyzed in autopsy studies of human infants conducted in Australia (Makrides et al., 1994) and the United Kingdom (Farquharson et al., 1995). Both studies found that the brain tissue of breastfed infants contained higher concentrations of DHA compared to their standard formula-fed counterparts. These findings are consistent with animal studies, where dietary restriction of *n-3* PUFA decreased the amount of DHA within the brain (Diau et al., 2005; Brenna, 2011; Luchtman and Song, 2013). One of the compelling arguments for including DHA and other LC-PUFAs in infant formula is to render its composition more similar to breast milk, which is commonly cited as the “gold standard” for infant nutrition (Burns et al., 2012). DHA enriched formula can enable formula-fed infants to attain DHA levels that are equivalent to their human milk receiving counterparts (Cunnane et al., 2000). However, clear functional benefits to infant development need to be demonstrated before DHA enriched formula can be unequivocally recommended.

## The functional effects of DHA on neurocognition: evidence from studies of deficiency in animals

The evidence that DHA deficiency is detrimental to neurocognitive development is well established in animal models. The functional consequences of lower DHA concentrations within the CNS in baboons include visual (Neuringer et al., 1986) and motor deficits (Champoux et al., 2002). Studies have found that *n*-3 PUFA deficient rodents exhibit poorer performance in the Morris water maze test compared to their *n*-3 PUFA-sufficient counterparts (Sheaff et al., 1999; Moriguchi et al., 2000; Lim et al., 2005). DHA deficient mice exhibit a range of neurocognitive impairments, including problems with learning and memory (Catalan et al., 2002) and can have a heightened stress response (Fedorova and Salem, 2006). Detailed reviews in this area have been conducted by Davis-Bruno and Tassinari (2011) and Luchtman and Song (2013).

Animal studies allow more flexibility with respect to study design and potentially offer greater insight into the neurological mechanisms influenced by DHA deficiency in human infants (Romijn et al., 1991). However, there are limitations in the extrapolation of findings obtained in animal studies to the study of nutrition in humans. Research undertaken in non-human primates offers certain advantages over rodent studies in terms of transferability to humans, related to similarities in relative brain size, retinal microarchitecture and other anatomical, physiological and genealogical homologies (Brenna, 2011). However, human neurocognitive functioning is more complex and relies to a greater degree on higher cognitive capacities such as language and executive functioning (Luchtman and Song, 2013). A degree of caution should be applied when generalizing findings from animals to humans (Innis, 2007). Nonetheless, there is a strong and consistent body of evidence obtained from animal studies linking *n*-3 LC-PUFA deficiency to impaired neurocognitive functions. These links warrant further investigation through clinical research in humans.

## Maternal dietary DHA intake and supplementation

Epidemiological studies of maternal DHA intake provide insight into the potential value of DHA on neurocognitive outcomes. A very large observational study (*n* = 11, 875) in pregnancy found a significant association between low maternal seafood consumption (<340 g per week) and suboptimal neurocognitive outcomes in childhood (Hibbeln et al., 2007). Children aged 6 months to 8 years whose mothers consumed low seafood diets during pregnancy had lower verbal IQ, displayed less pro-social behavior (defined as voluntary behavior intended to benefit another) and had poorer social and communication skills compared to those whose mothers consumed high seafood diets (Hibbeln et al., 2007). Furthermore, in a study of Canadian Inuit people (who typically consume a DHA-rich diet) DHA concentrations in umbilical cord plasma were positively associated with longer gestation, better visual acuity, higher scores of novelty preference on the Fagan test at 6 months and higher cognitive scores on the BSID-II of mental and psychomotor performance at 11 months (Jacobson et al., 2008). Most published observational epidemiological studies have recognized a positive association between maternal intake of *n*-3 LC-PUFA rich foods during pregnancy and neurocognitive development of offspring (Oken et al., 2005, 2008; Hibbeln et al., 2007; Mendez et al., 2009; Boucher et al., 2011). The observational study by Gale et al. (2008), however, detected no association between the frequency with which mothers ate fish in pregnancy and full-scale or performance IQ of their offspring at 9 years of age. Nevertheless, these researchers did find that children whose mothers had eaten oily fish had higher verbal IQ and a reduced risk of hyperactivity compared to those children whose mothers did not eat oily fish, after adjustment for potential confounding factors. However, the findings of these studies are somewhat weakened due to potentially confounding variables, including social and economic differences that may independently affect neurodevelopmental outcomes in childhood (Drane and Logemann, 2000; Boyd et al., 2013).

It is well established that maternal fish oil supplementation during pregnancy substantially increases fetal DHA concentration at the time of birth (Larqué et al., 2012). Furthermore, two randomized controlled trials (RCTs) have shown that DHA supplementation during pregnancy offers significant benefit to infant neurocognitive development (Judge et al., 2007; Dunstan et al., 2008). However, the RCTs in this area are not easily comparable and positive effects have not been identified in all studies (Lo et al., 2012). One long term follow-up found that maternal supplementation enhanced neurocognitive outcomes up to 4 years later (Helland et al., 2003) but the effect did not persist after 7 years (Helland et al., 2008). A relatively large RCT (*n* = 249) from Bangladesh found that maternal DHA supplementation from 25 weeks gestation until delivery had no effect on infant BSID-II mental and psychomotor performance outcomes at 10 months (Tofail et al., 2006). Such findings may not be directly comparable to an Australian population since maternal nutrition and anthropometric status in developing countries are often low (Karim and Mascie-Taylor, 1997; Dhar et al., 2003). It is also possible that the control oil used in this study (which contained α-LA: 2700 mg and LA: 2250 mg per day) may have inadvertently promoted infant neurocognitive status, thereby attenuating any treatment effect from the intervention (Tofail et al., 2006).

A meta-analysis and systematic review of maternal *n*-3 LC-PUFA supplementation undertaken by Gould et al. (2013) highlighted numerous potential areas of bias in the current literature and remarked on the relatively poor quality of most RCTs in this field. Of major concern was the consideration that publications seldom reported the randomization process and/or method(s) used to conceal treatment allocation from participants. According to this review, the only RCT of maternal DHA supplementation and infant neurocognitive outcomes that was considered to be genuinely free from bias was conducted in Australia by Makrides et al. (2011a). This RCT found no significant association between moderate DHA supplementation (800 mg per day) during pregnancy and BSID-II scores of language or cognitive development at 2½years (*n* = 2399). However, this RCT revealed that children in the treatment group manifested significantly lower incidence of cognitive delay compared to their un-supplemented counterparts.

In summary, RCTs and epidemiological studies evaluating the potential neurocognitive impact of maternal DHA supplementation during pregnancy have revealed somewhat heterogeneous results in healthy term infants. Consequently, it may be premature to make unequivocal recommendations about any neurocognitive benefits of DHA supplementation in healthy term infants based on the currently available research findings.

## Correlations between infant neurocognition and breastfeeding

Over the years, many prospective observational studies have indicated that breastfed infants have a significant neurocognitive advantage over their formula fed counterparts (Anderson et al., 1999; Agostoni et al., 2001; Oddy et al., 2003, 2011; Kramer et al., 2008). It has been theorized that this is due to the higher presence of DHA in breast milk, relative to formula milks. Some studies have found positive associations between DHA concentrations within breast milk and/or infant blood levels and better outcomes of visual acuity (Innis et al., 2001; Jørgensen et al., 2001). However, it is likely that breastfeeding enhances infant development due to a number of inter-related factors as reviewed in Jain et al. (2002). Specifically, observational studies are potentially confounded by the heterogeneous composition of breast milk (both within and between lactating individuals), environmental factors such as maternal/infant bonding and other influences (Jain et al., 2002). One particularly significant potentially confounding factor is social economic status (SES), which is positively associated with both maternal and infant IQ, along with the decision to breastfeed (Der et al., 2006; de Jager et al., 2013). Furthermore, the breastfeeding act itself may be indicative of maternal attentiveness and nurturing which may independently foster long term positive effects on infant neurocognitive outcomes (Morley et al., 1988). Despite appropriate statistical techniques used to try to control for the influence of confounders, observational studies can be subject to systematic bias and results should therefore be interpreted with some caution.

A study by Caspi et al. (2007) tested the association between breastfeeding and child IQ with respect to FADS2 genetic profile, specifically in terms of the SNP rs174575. Breastfed infants who were rs174575 C-dominant carriers achieved higher scores on standardized IQ tests compared to the C-carriers who were not breastfed. Meanwhile, children homozygous for the minor allele (GG genotype) were found to have similar IQs, irrespective of feeding method. These findings remained statistically significant after accounting for potential confounding variables including intrauterine growth, family social economic status, and maternal cognitive ability. While potentially important in terms of the possible interaction between breastfeeding and the genetic status of the infant, these findings have yet to be replicated by other research studies.

## Correlations between infant neurocognition and DHA status

Many trials have found that higher plasma or RBC DHA concentrations (often as a result of LC-PUFA supplementation) are positively correlated with infant neurocognitive outcomes (Agostoni et al., 1995, 1997; Gibson et al., 1997; Birch et al., 2000; Innis et al., 2001; Helland et al., 2003; Innis, 2003; Jensen et al., 2005; Drover et al., 2011). Some studies, on the other hand, have found no significant relationships (Lucas et al., 1999; Makrides et al., 2000; Auestad et al., 2001), while conversely two studies have found that higher infant DHA blood concentration has a negative neurocognitive effect (Scott et al., 1998; Lauritzen et al., 2005). It should be cautioned that associations between DHA status and infant neurocognitive status do not necessarily demonstrate causality, nor do they demonstrate the effectiveness of the intervention alone—as this may be confounded by other nutrients (Innis, 2003).

## RCTs of LC-PUFA supplementation in term and preterm populations

In order to confirm whether dietary LC-PUFA is responsible for the enhanced neurocognitive outcomes associated with breastfeeding and higher DHA status, RCTs of LC-PUFA supplementation are necessary. Several of these trials have been conducted, usually in formula-fed infants. The most common methodology involves comparing the neurodevelopmental outcomes of infants randomized to receive infant formula with DHA (either alone or in combination with AA and/or other PUFAs) or placebo (un-supplemented formula). The majority of trials in healthy term infants have shown little or no consistent, beneficial effects on neurocognitive outcomes as a result of dietary LC-PUFA supplementation. However, infant LC-PUFA supplementation has resulted in no negative effects on growth, development or morbidity (Koletzko et al., 2005). There is, therefore, currently no compelling argument either for or against LC-PUFA supplementation in term infants with respect to neurocognitive outcomes. This conclusion has been re-iterated in three consecutive versions of the Cochrane review (Simmer, 2001; Simmer et al., 2008, 2011) that have evaluated 9, 14, and 15 relevant RCTs, respectively. Interestingly, in the authors' conclusions for both the (2008) and (2011) Cochrane reviews, Simmer et al. refer to the positive results found by the Dallas group (Birch et al., 2005) and state that these results need to be replicated in other settings. These authors also propose that future RCTs should explore the use of DHA derived from single cell microalgae and supply higher doses of DHA, in line with typical human milk (DHA 0.32%) concentrations throughout the world.

Hoffman et al. (2009) reviewed 20 RCTs within this field including several studies not included in the Simmer et al. (2008) Cochrane review and elaborated on some methodological factors including dosage, source, and duration of supplementation. Hoffman et al. (2009) concluded that trials which supplied term infants with DHA in concentrations greater than 0.3%TFA (in addition to AA >0.3%TFA) were more likely to identify a significantly positive effect on neurocognitive and visual outcomes. In another meta-analysis utilizing individual patient data (IPD) with a considerable sample size (*n* = 870), Beyerlein et al. (2010) combined the raw scores from four methodologically similar RCTs (Lucas et al., 1999; Fewtrell et al., 2002, 2004; Bouwstra et al., 2005) which each assessed BSID-II outcomes at 18 months. The analysis concluded that LC-PUFA supplemented formula conveys no significant benefit on neurodevelopment at 18 months, as assessed using the BSID-II. However, this meta-analysis was unable to access IPD from all relevant trials (Birch et al., 2000; Clandinin et al., 2005).

Evidence that infant DHA supplementation conveys significant benefit on visual acuity is derived from a systematic review of 12 clinical studies from the Harvard School of Public Health (SanGiovanni et al., 2000). SanGiovanni et al. (2000) incorporated the results from both randomized and non-randomized studies of DHA supplemented formula and concluded that increased dietary DHA improved visual acuity in term infants at two and four months of age. It should be cautioned that analyses that combine findings from both randomized and non-randomized trials have a higher risk of incorporating selection bias in the recruitment for the trial (Szajewska, 2011). More recent reviews in this field suggest that more research is required before definitive recommendations can be made concerning whether term infants would benefit from *n*-3 LC-PUFA supplemented formula (Agostoni, 2008; Benton, 2008; Belkind-Gerson et al., 2008; Makrides et al., 2011b; Campoy et al., 2012).

The majority of RCTs of infant DHA supplementation (as described above) use infant formula and are constrained by the consideration that their study samples have chosen not to breastfeed, thereby reducing external validity of the findings (Gibson and Makrides, 1998; Smithers et al., 2008a). There have been a small number of studies which have supplemented infants with DHA directly, thereby bypassing the need to employ formula-based supplementation. To the best of our knowledge, only one such direct supplementation RCT has addressed the effect of DHA on infant neurocognitive functioning (Meldrum et al., 2012). In this recent double-blinded, placebo-controlled trial conducted in Australia (Meldrum et al., 2012), healthy term infants (*n* = 287) were randomized to either very high dose fish oil (incorporating >250 mg DHA plus 60 mg EPA) or placebo (olive oil) per day from birth to 6 months. The study determined that while infants within the fish oil group had significantly higher DHA concentrations in erythrocyte and plasma phospholipids at 6 months of age relative to the placebo group, there was no significant difference between standard or composite scores of the BSID-III (third edition) at 18 months or on outcomes from the Child Behavior Checklist. In a subtest which explored the development of infant communication skills (*n* = 185), the study found that scores for later developing gestures and total number of gestures were significantly higher in the fish oil group compared to the placebo group at both 12 and 18 months. This finding is interesting since gestural skills in infancy are associated with visual recognition memory, deferred imitation and turn-taking skills (Heimann et al., 2006). Furthermore, these skills are understood to predict language and communicative ability in later life (Acredolo and Goodwyn, 1988). Direct supplementation of the oil emulsion allowed participation of breast- and/or formula-fed infants alike. However, a potential criticism of this study is that the odor of the fish oil may not have been adequately masked, and therefore parents were frequently able determine which treatment their child was receiving. This, in turn, may have affected parents' ratings of their child's gestural abilities.

While the focus of the current paper is on term infant neurocognitive response to DHA supplementation, valuable information can be gleaned from investigation into the preterm population. Preterm infants are especially vulnerable to DHA deficiency as they have not had access to maternal lipid stores for the normal period of gestation (Haggarty, 2002). Similar to full term infants, preterm infants fed formula milk without DHA have lower DHA status compared to those fed human milk (Carlson et al., 1986) or LC-PUFA supplemented formula (Koletzko et al., 1989; Lapillonne et al., 2000). It has been identified that children born preterm have higher rates of learning disabilities, language impairment, attention deficits, hyperactivity, and reduced cognitive test scores compared to (gender- and age-matched) children born at term (Bhutta et al., 2002; Perricone and Morales, 2011). Although there are many factors associated with preterm delivery, it is possible that lower DHA status during the critical period of brain growth may contribute toward impaired neurocognitive development (McNamara and Carlson, 2006).

The DINO (DHA infant neurodevelopmental outcomes) double-blind RCT provided supplementation to lactating mothers (*n* = 657) in order to increase breast milk DHA (Makrides et al., 2009). Lactating women were either supplied six 500 mg DHA-rich tuna oil capsules per day or placebo soy oil capsules. Mothers were encouraged to breastfeed; however, if the mother chose not to provide breast-milk or if additional milk was required, infant formula was provided. The DHA concentrations of the formula matched the typical milk DHA concentrations of the two groups. The study found that preterm infants in the DHA group had better visual development (as determined through sweep VEP acuity) at 4 months corrected age [i.e., chronological age corrected for the degree of prematurity; (Smithers et al., 2008b)]. A higher mean mental development index of children in the DHA supplemented group (as assessed using the BSID-II) was found. However, after adjusting for confounding factors this benefit was not statistically significant (*p* = 0.2). Yet the number of children in the DHA group with low cognitive scores (indicative of mildly delayed development) was significantly smaller in the treated group compared with the control group. In pre-planned secondary analyses, the DINO study found DHA supplementation had a significantly positive effect on cognitive outcomes in girls compared to boys. The reason for differences in response to DHA as a function of gender remains unclear but may be related to the higher rate of LC-PUFA synthesis that has been identified in females (Burdge and Nagura, 2002). The authors proposed that the dose of DHA chosen for this study may not have been sufficient to elicit equivalent neurocognitive benefits in males and they considered whether enhancing DHA concentrations may evoke neurocognitive advantages (Makrides et al., 2009). Furthermore, the authors concluded that the DHA concentration in standard human breast milk of Australian women is sub-optimal for the visual development of preterm infants (Smithers et al., 2008b).

There have been numerous LC-PUFA supplementation trials in preterm infants which have reported greater visual acuity following enhanced dietary DHA relative to placebo (Birch et al., 1992; Carlson et al., 1993; Smithers et al., 2008b). Similarly, numerous RCTs in preterm populations have found that DHA supplementation positively affects neurocognitive outcomes including language comprehension (O'Connor et al., 2001), memory (Henriksen et al., 2008) and mental and psychomotor development (Clandinin et al., 2005). However, the review by Qawasmi et al. (2012) points out that there are also many RCTs which have consistently shown no neurocognitive effect, both in preterm and term infant populations. Similarly, Schulzke et al. (2011) concluded that there is insufficient evidence to recommend DHA supplementation with respect to routine neonatal care in preterm infants. The most recent Cochrane review of LC-PUFA supplementation of preterm formula concludes that there are no clear long-term benefits on visual or intellectual development after pooling data across studies of preterm infants (Schulzke et al., 2011). Finally, two recently published reviews reflect uncertainty as to whether preterm infants should be supplemented with dietary DHA with respect to potential long term neurocognitive or developmental benefits (Molloy et al., 2012; Lapillonne et al., 2013). Further investigation is clearly warranted concerning the role of DHA supplementation in modulating optimal neurocognitive outcomes in preterm infant populations.

## Genetic factors modulating individual dietary requirements for *n*-3 LC-PUFAs

A relatively new area of investigation concerns how genetic differences may modulate individual LC-PUFA requirements. Such research has focused on the genes FADS1 and FADS2. These genes are known to act upon the enzymes delta-5 desaturase (D5D) and delta-6 desaturase (D6D) and influence the efficiency with which shorter chain *n*-3 and *n*-6 PUFAs are converted into LC-PUFA products (Nakamura and Nara, 2004; Schaeffer et al., 2006; Rzehak et al., 2009; Bokor et al., 2010; Glaser et al., 2011). The D5D and D6D enzymes are present in the human liver from early gestation (Innis, 2005). The fetus is capable of synthesizing *n*-3 LC-PUFAs from shorter chain *n*-3 precursors from 26 weeks gestation (Uauy et al., 2000).

SNPs within FADS1 and FADS2 have been associated with the ratio of desaturation precursors (i.e., α-LA, LA, eicosadienoic acid (EDA; 20:2 *n*-6) and dihomogamma linolenic acid (DGLA; 20:3 *n*-6) to desaturation products, including the LC-PUFAs (EPA, DPA, and AA) (Schaeffer et al., 2006; Gillingham et al., 2013). In a very large German study conducted in an adult population, the authors found that up to 28% of AA variability was associated with 11 common SNPs (and 5 SNPs of reconstructed haplotypes) from the FADS1 and FADS2 gene clusters (Schaeffer et al., 2006). Yet, the FADS polymorphisms tested by Schaeffer et al. (2006) could only explain ~7% of EPA and 3% of DHA levels. Few subsequent studies have been able to identify a significant association between FADS polymorphisms and DHA concentrations in human populations (Koletzko et al., 2011; Lattka et al., 2013). However, Koletzko et al. (2011) identified significant associations between fetal and maternal FADS genotypes and DHA concentrations in fetal circulation, irrespective of maternal diet. While it currently appears that DHA concentrations are primarily modulated though dietary supply, several FADS genetic polymorphisms are still under investigation internationally (Glaser et al., 2011; Gillingham et al., 2013) and are currently being considered in our laboratory.

## Interpreting current research findings

Inconsistent findings within this literature have been the subject of much consideration. suggestions as to why discrepancies may have occurred are outlined in the review published by Meldrum et al. (2011). Previous studies were identified as having considerable variability in: (i) inadequate sample sizes, (ii) doses of DHA utilized, (iii) source of DHA used (i.e., algal or fish sources), (iv) age at which supplementation was initiated, (v) duration of supplementation, (vi) type of neurocognitive/developmental assessments used to evaluate neurocognitive functioning, and (vii) variability in participant compliance across studies. It was also speculated in this review that genetic polymorphisms might represent a potentially relevant factor affecting the outcomes of these RCTs. It is now recognized that dietary requirements for DHA and other LC-PUFAs may be somewhat heterogeneous across individuals; for example, being somewhat dependent on an individual's genetic profile (Koletzko et al., 2011).

In addition to FADS genetic considerations, the lack of consistency across RCTs of DHA supplementation may also be due, at least in part, to gender based differences in LC-PUFA metabolism from shorter chain precursors (Guesnet and Alessandri, 2011). It is known that the capacity to convert α-LA into *n*-3 LC-PUFAs including DHA is significantly greater in females than in males (Burdge, 2004), resulting in higher DHA circulating plasma concentration in females (Giltay et al., 2004). This is thought to be due to the influence of estrogen and other hormones on the activity and expression of D5D and D6D in the liver (Extier et al., 2010; Decsi and Kennedy, 2011). Evidence from the Western Australian pregnancy cohort (Raine) study (*n* = 1038) has also found long term neurocognitive benefits from breastfeeding to be gender specific (Oddy et al., 2011). This study found that breastfeeding had a more pronounced neurocognitive benefit on male infants, as evident until 10 years of age (Oddy et al., 2011). A gender specific response to DHA enriched human breast milk was also observed by Makrides et al. (2009) in the aforementioned DINO study of preterm infants. Females manifest a greater capacity for LC-PUFA synthesis (Burdge and Nagura, 2002), such that males may have higher dietary DHA requirements during infancy (Makrides et al., 2009).

## Conclusions and future directions

DHA is known to play a critical role in the developing human brain and there is evidence of its neurobiological importance during infancy (McCann and Ames, 2005). DHA deficiencies in animals have proven to exert deleterious effects on a range of neurodevelopmental outcomes (McNamara and Carlson, 2006). High intake of oily fish during pregnancy appears to benefit children's neurocognitive development. Additionally, several studies have demonstrated positive associations between infant blood concentrations of DHA and neurocognitive status (Innis, 2003, 2007). Yet previous RCTs undertaken in this field provide conflicting evidence concerning the putative neurocognitive benefits of *n*-3 LC-PUFA supplementation in healthy full term infants. Despite the consideration that the majority of RCTs report little or no effect from supplementation (as cited in several Cochrane reviews and meta-analyses), many researchers suggest that further work should be undertaken in order to better define optimal DHA intakes before and during infancy.

It is possible that DHA deficiency during critical periods of brain growth and structural organization may render the brain vulnerable to neurological or neurodegenerative diseases later in life (Farquharson et al., 1995). The development of the brain's architecture prenatally and during infancy lays down the foundation on which the structure of the adult brain is based. It is therefore possible that early modification of LC-PUFA levels will have long-term structural and functional consequences which may be too long-term and/or subtle to detect in healthy infants and children (McNamara and Carlson, 2006). However, more noticeable effects may emerge in older individuals. In this context, the equivocal findings obtained in this field to date are heuristic, as they will stimulate further research which should more rigorously control for the potentially confounding variables that have been identified in this review. As proposed by Alderson and Roberts (2000), inconclusive results are potentially very informative in biomedical science as they stimulate further inquiry and ultimately improve health outcomes for future generations.

While RCTs are generally thought to provide the most robust indexes of causal mechanisms in human clinical research (Szajewska, 2011), the results from observational studies in humans and intervention studies in laboratory animals also deserve serious consideration. In future, larger and higher dose RCTs should be undertaken using more sensitive measures of infant and child neurocognitive capacity. A more careful approach to the design and analysis of observational epidemiological studies is also likely to yield tangible benefit (for example, through undertaking larger studies which are able to consider a wider range of potentially confound variables).

Debate about the usefulness of dietary DHA during infancy has been ongoing for over 30 years and a wealth of data have been collected by research groups around the world. It would be of far-reaching benefit for more international collaboration to be undertaken within this area of inquiry. Data access facilitated through computational platform/s that enable sharing of de-identified, IPD could provide researchers with the means to carry out statistically novel methods of data interpretation on a very large scale. Meta-analysis of IPD allows researchers to analyze the raw data of relevant original studies (using similar covariates) and thereby derive potentially more reliable conclusions (Stewart and Parmar, 1993). To the best of our knowledge, only one IPD meta-analysis on the neurocognitive effects of LC-PUFA supplementation during infancy has been conducted (Beyerlein et al., 2010). This meta-analysis was able to access the raw data from 4 out of the 6 relevant RCTs. The limitations imposed on this IPD meta-analysis by the non-availability of data from two relevant RCTs could be prevented in future if guidelines and policies are implemented within a collaborative international scientific framework that is more sensitive to ethico-legal and data-ownership issues.

Despite the absence of a scientific consensus with regard to putative benefits (as identified in this review), many manufacturers of infant formula include DHA and AA in certain formulations and market them as “superior products” which provide a distinct neurocognitive advantage (Simmer et al., 2011). This shaping of public opinion through retail is controversial, considering that there is little concrete scientific evidence to support these claims. Australia is one of the leading markets for *n*-3 products and supplements and, consequently, consumers are at increased risk of being misled by current marketing approaches (McManus et al., 2011). It is important for future research to address these claims definitively, so as to either avoid unnecessary supplementation of infants' diets (and the associated economic cost) or to ensure that infants are universally provided with adequate dietary DHA to prevent suboptimal neurocognitive development.

### Conflict of interest statement

The authors declare that the research was conducted in the absence of any commercial or financial relationships that could be construed as a potential conflict of interest.
